# Deadly Wells: Taking Action to Protect Future Generations

**Published:** 2006-09

**Authors:** Mahfuzar Rahman

**Affiliations:** Guest Editor, Public Health Sciences Division, ICDDR,B, GPO Box 128, Dhaka 1000, Bangladesh, Email: mahfuzar@icddrb.org, Fax: +880-2-886050

Children around the world are now confronting serious health risks from environmental hazards that were neither known nor suspected before. Over 40% of the global burden of disease attributed to environmental factors falls on children aged less than five years. Thousands of chemical compounds have been developed over the last 50 years. Considering economic and social development, proper actions need to be taken to allow children to grow up and develop in good health.

## Studying children with arsenic exposure

One in thirty people of the world is chronically exposed to inorganic arsenic, a class I human carcinogen (1), and millions of children of the world are currently exposed to arsenic-contaminated drinking-water. Many more are exposed during their in utero-life. These uncertainties of exposure in early life may pose an unknown health risk, which has lifelong implications.

## Arsenic and neonatal, infant and child health

Children sometimes have different susceptibility to hazardous chemicals than adults. Recent reports suggest that inorganic arsenic also poses increased reproductive and developmental risks; however, relatively little is known about trans-placental exposure (1–3). Limited information is available on the possible health effects of arsenic among exposed children, despite the fact that arsenic readily passes the human placenta. It is already documented that high exposure to arsenic causes detrimental effects on the developing foetus (1–3).

Earlier studies have focused only on adult populations. Clinical symptoms now have also been diagnosed in children. In addition to arsenic-specific hyperkeratosis and pigment disorders, other adverse effects of arsenic exposure on children include cognitive delays, reduced IQ, mental slowing, and poor memory. High susceptibility of children is probably due to higher arsenic exposure per body-weight compared to adults. Chronic exposure to arsenic is related to low IQ, child retardation, and low intellectual ability (4). However, malnutrition is common in developing countries, which could have an influence on child health, especially verbal abilities and long-term memory.

A recent study has shown that arsenic in drinking-water during early childhood or in utero-life causes malignant and non-malignant lung diseases and subsequently increased mortality among young adults (5). Lung cancer is actually the main long-term cause of death due to chronic exposure to arsenic (1).

## Is the future generation at risk?

How chronic exposure to arsenic affects children is still unknown. Children are likely to be more susceptible to arsenic than are adults. Organ maturation usually takes place during early childhood. The possible impact of arsenic on child health has not yet received sufficient attention, but the collective evidences suggest a large potential for adverse effects on child health and development. Long-term effects of arsenic exposure, particularly its effects on children, need to be investigated. A clear understanding is needed to protect children's health and their health conditions in later years. The public-health impact of adverse effects relating to arsenic exposure in early life could be substantial given the large number of children exposed worldwide.

## Public-health responses to combat the situation

Public education needs to be well-designed and imparted in an appropriate manner regarding the issues and child health risk-reduction options. Effective information channels are, therefore, needed (6, 7). Public-media campaigns will require coordination between the public and the private sector to ensure the quality, coverage, and regular update of information. However, the priority must be given to selecting safe drinking-water alternatives.

The activities implemented to increase awareness have been successful and helped raise the awareness level of the community about the arsenic problem and the different mitigation options that are available. Estimating the burden of disease associated with each technology described in this issue (8) will generate new knowledge on the provision of selecting safe drinking-water, their technical viability, and community acceptance. Various sources of safe drinking-water should be considered in future research considering their ease of use, low cost, and simplicity.

## Taking action to protect children from further exposure

Several arsenic-mitigation options have been described in this issue, e.g. removal of arsenic from groundwater (9, 10), controlling human pathogens from surface water (11), and provision of improved dugwells (12). These options are economically and culturally challenging, but particularly thought on a large scale. Based on the quantitative evidence presented in this issue, it appears that testing and monitoring of wells managed at the village level combined with judicious installation of low-arsenic community wells in high exposure areas (13) could rapidly reduce arsenic exposure at the national scale. However, it is essential to evaluate further the contribution of various foods to the total arsenic exposure as described in this issue (14). The public-health impact of other sources (exposure via food-chain) is largely unknown as epidemiologic focus has only been exposure via drinking-water. Further sources of exposure are needed for future epidemiological studies. Temporal variations in arsenic concentrations in wells are described in this issue (15, 16). However, further investigations are needed at this end for monitoring well-water at regular intervals.

Safe water is needed to protect the health of foetuses, infants, and young children. Considering the healthy environment of the children, every level has a role to play from the members of the family and community to local, regional, national and international bodies. As millions of children and women are chronically exposed to arsenic in Asia, the potential impacts of arsenic exposure on children and their future life are an urgent problem confronted by countries most affected by arsenicosis. The public-health responses must be addressed in an integrated, comprehensive approach to mitigate the sufferings of the arsenic-affected young population and future generation.
